# Inequity in catastrophic costs among tuberculosis-affected households in China

**DOI:** 10.1186/s40249-019-0564-2

**Published:** 2019-06-19

**Authors:** Cai-Hong Xu, Kathiresan Jeyashree, Hemant Deepak Shewade, Yin-Yin Xia, Li-Xia Wang, Yan Liu, Hui Zhang, Li Wang

**Affiliations:** 1 National Center for Tuberculosis Control and Prevention, Chinese Center for Disease Control and Prevention, Beijing, 100226 China; 2 Velammal Medical College Hospital and Research Institute, Madurai, 625009 India; 3 International Union Against Tuberculosis and Lung Disease (The Union), South-East Asia Office, New Delhi, 110016 India; 4 International Union Against Tuberculosis and Lung Disease (The Union), 75006 Paris, France; 5 Karuna Trust, Bengaluru, 560041 India; 6 Department of Epidemiology and Biostatistics, Institute of Basic Medical Sciences Chinese Academy of Medical Sciences, School of Basic Medicine Peking Union Medical College, Beijing, 100005 China

**Keywords:** Catastrophic health expenditure, tuberculosis, Patient cost, Universal health coverage, Social protection, Equity

## Abstract

**Background:**

There are limited nationally representative studies globally in the post-2015 END tuberculosis (TB) era regarding wealth related inequity in the distribution of catastrophic costs due to TB care. Under the Chinese national tuberculosis programme setting, we aimed to assess extent of equity in distribution of total TB care costs (pre-treatment, treatment and overall) and costs as a proportion of annual household income (AHI), and describe and compare equity in distribution of catastrophic costs (pre-treatment, treatment and overall) across population sub-groups.

**Methods:**

Analytical cross-sectional study using data from national TB patient cost survey carried out in 22 counties from six provinces in China in 2017. Drug-susceptible pulmonary TB registered under programme, who had received at least 2 weeks of intensive phase therapy were included. Equity was depicted using concentration curves and concentration indices were compared using dominance test.

**Results:**

Of 1147 patients, the median cost of pre-treatment, treatment and overall care, were USD 283.5, USD 413.1 and USD 965.5, respectively. Richer quintiles incurred significantly higher pre-treatment and treatment costs compared to poorer quintiles. The distribution of costs as a proportion of AHI and catastrophic costs were significantly pro-poor overall as well as during pre-treatment and treatment phase. All the concentration curves for catastrophic costs (due to pre-treatment, treatment and overall care) stratified by region (east, middle and west), area of residence (urban, rural) and type of insurance (new rural co-operative medical system [NCMS], non-NCMS) also exhibited a pro-poor pattern with statistically significant (*P* <  0.01) concentration indices. The pro-poor distribution of the catastrophic costs due to TB treatment was significantly more inequitable among rural, compared to urban patients, and NCMS compared to non-NCMS beneficiaries.

**Conclusions:**

There is inequity in the distribution of catastrophic costs due to TB care. Universal health coverage, social protection strategies complemented by quality TB care is vital to reduce inequitable distribution of catastrophic costs due to TB care in China.

**Electronic supplementary material:**

The online version of this article (10.1186/s40249-019-0564-2) contains supplementary material, which is available to authorized users.

## Multilingual abstracts

Please see Additional file 1 for translations of the abstract into the five official working languages of the United Nations.

## Background

The World Health Organization (WHO) issued a post-2015 global tuberculosis (TB) strategy that envisaged “a world free of TB” with zero death, disease, and suffering due to TB by 2035. One of its four principles is to ensure protection and promotion of human rights, ethics and equity [1]. This is in line with the policy to move health systems closer to universal health coverage, which is conventionally defined as access to health care without risk of financial hardship due to out-of-pocket health care expenditures [2]. Besides free or affordable TB care, social protection interventions are required that prevent or mitigate other financial risks associated with TB. This is also vital to attain the sustainable development goals [3].

TB is mainly a disease of the poor and marginalized people and communities [4]. TB affects the poorest segment of society disproportionately and thus the impoverishing effects of TB are gravest for those who are already vulnerable [2, 3]. Though it is quite likely that distribution of catastrophic costs is pro-poor, there are limited nationally representative studies globally in the post-2015 period regarding documentation of wealth related inequity in the distribution of catastrophic costs due to TB care. In India (during TB diagnosis in 18 randomly selected districts in 2016–2017) [5] and China (during TB treatment in six counties in 2013) [6], catastrophic costs was disproportionately high among the poorest quintile.

China conducted a nationally representative “TB patient cost survey” in 2017 [4]. This study reports the extent of equity in distribution of TB care costs (pre-treatment, treatment and overall), total costs as a proportion of the annual household income (AHI) and catastrophic costs due to TB, also compared across regions, residence and insurance schemes.

## Methods

### Design

This was a cross-sectional analytic study involving primary data collection.

### Setting

#### Health financing in China

China is a developing country with a per capita gross national product of USD 7941 in 2016. The total expenditure on health in 2016 was USD 6815 billion, accounting for 6 % of the gross national product [7]. The provinces are divided into east, middle and west region. Three percent of people fall below the poverty line (USD 430) [7]. The health care delivery system is “mixed” with a dominant role for public sector institutions [8].

Public funded health insurance schemes cover more than 95% of the population. There are three basic schemes namely urban employee basic medical insurance (UEBMI), urban resident basic medical insurance (URBMI), and new rural cooperative medical scheme (NCMS). Payroll taxes are the main funding source for UEBMI and government subsidies are the major funding sources for NCMS and URBMI. NCMS funds are pooled at the county level and URBMI and UEBMI are pooled at the prefecture level. The benefit packages and financial protection are not equal within and across the schemes, which is a crucial barrier to achieving universal health coverage in China. The service package of NCMS was smaller and the reimbursement level was 10% lower than URBMI or UEBMI [9].

#### China national TB Programme (NTP)

The National center for tuberculosis control and prevention, which belongs to China Centre for Disease Control (CDC), is in charge of NTP. TB management units are established at provincial, prefecture and county levels (basic management units [BMU] at county level). TB diagnostic facilities are centralized at the BMU level and rarely available at township level (below county). Diagnosed patients are registered in web-based TB information management system (TBIMS) and initiated on directly observed therapy (DOT) at BMU with assistance from township clinics and village health workers.

Nearly 90% of the patients with TB get treatment within these designated facilities. TB patients are provided free chest radiography, free sputum smear test and free first-line drugs. Additional TB services in the form of other investigation and ancillary drugs are charged.

### Study population

Drug susceptible pulmonary TB patients who had received at least 2 weeks of intensive phase therapy at the time of national TB patient cost survey (March to June 2017) were included. Pulmonary TB included pediatric TB and TB with comorbidity. We excluded people who were treated in facilities not under NTP.

#### Sample size

Assuming the prevalence of catastrophic costs due to TB was 30% [10], relative precision as 0.2 and α error as 0.05, average cluster (defined at county level) size of 50, between-cluster variation of 0.4, design effect of 4.36 and anticipating a non-response rate of 10%, the final sample size was 1086, to be sampled from 22 clusters (see Additional file 2: Annex 1).

#### Sampling methodology

We adopted multi-stage stratified cluster sampling. There were significant variations in the economy and the TB prevalence across China. The per capita gross national product of the six provinces sampled under the survey is shown in Additional file 2: Annex 2. The main stratifying factors were patient’s region and residence (rural/urban one each from each of the east/middle/west provinces - see Additional file 2: Annex 3 for the steps followed in sampling)**.** The 22 counties included in the study are depicted in Additional file 2: Annex 4.

### Data collection and management

#### Data collection

Face to face interview (at BMU in the county) was done by trained investigators (trained university students and staff from China CDC) using a structured questionnaire (see Additional file 2: Annex 5). Costs related information was collected from symptom onset up to the day of interview. Direct medical costs included the costs for outpatient registration, hospitalization, investigations and medicines. Direct non-medical costs included transportation, accommodation and food of the patients and family members. Indirect costs were estimated as the total period of absence from work in hours multiplied by the hourly wage of the absent worker. The investigators directly asked the annual income of the patients.

#### Data management and analysis

Data were double entered and validated using EpiData 3.1 (EpiData Association, Odense, Denmark) during July to December 2017. The analysis was conducted using STATA 12.1 (copyright 1985–2011, StataCorp LP, Texas USA).

We calculated the average monthly direct medical cost, direct non-medical costs and indirect costs during treatment. This average was used to impute the treatment costs of patients within the county for the remainder of treatment (assuming a total of 6 months for new patients and 8 months for previously treated patients).

The analysis was done separately for the pre-TB treatment phase, treatment phase and TB care overall (pre-TB treatment and treatment phase combined). Costs were described using the median and inter quartile range (IQR). The total costs (direct medical, direct non-medical and indirect costs combined) were defined as catastrophic if they exceeded 20% of pre-TB annual household income [4].

Income quintiles were generated by ranking the households based on monthly income per capita (MIPC). The distribution of total costs due to TB care were summarized across income quintiles as follows: i) absolute total costs, ii) annual total costs as a proportion of pre-TB AHI, and iii) proportion of households experiencing catastrophic costs.

Concentration curves and concentration indices (along with 95% confidence intervals [*CI*]) were used to assess the extent of equity in the distribution of all the above three indicators. The concentration curves plot the cumulative distribution of the health outcome variable in the y axis against cumulatively ranked households (poorest to richest) on the x axis. The values of concentration index ranges from + 1 to − 1; with positive value (concentration curve below the line of equality) suggesting pro-rich and negative value (concentration curve above the line of equality) suggesting a pro-poor distribution [11, 12].

For the indicator ‘total costs’, we assumed equity if the concentration curve and index revealed significant distribution across the richest quintiles (positive concentration index, 95% *CI* not including zero). For the indicators, ‘annual total costs as a proportion of pre-TB AHI’ and ‘catastrophic costs’, we assumed equity if the concentration curve and index revealed equal distribution across the quintiles (concentration curve not significantly different from the line of equality). The statistical significance of the concentration index was interpreted based on whether or not its 95% *CI* included zero.

We also compared the concentration curves across various subgroups (insurance type, residence and region) using dominance tests [11]. For further details on analysis, the readers may refer to these references by Demery L, McIntyre D et al. and O’Donnell O et al. [5, 11, 12].

## Results

### Demographic and socio-economic profile of patients

Of 1147 TB patients, 811 (70.7%) were male and mean age was 51 years (range 12–89). A total of 422 (36.8%) patients came from east region, 322 (28.1%) from middle region and 403 (35.1%) from west region. Sixty five percent patients resided in rural areas. NCMS covered 864 (75.3%) of the patients. The median (IQR) MIPC was USD 190 (46, 243). The incomes of 223 (19.4%) households were below the poverty line (Table 1).

**Table 1:** Demographic and socio-economic profile of patients enrolled in China’s TB^a^ patient cost survey (2017) (*n =* 1147)

Variable	*n*	(%)
Total	1147	(100)
Age group in years		
< 15	6	(0.5)
15–44	352	(30.7)
45–64	497	(43.3)
≥ 65	292	(25.5)
Gender		
Male	811	(70.7)
Female	336	(29.3)
Region		
East	422	(36.8)
Middle	322	(28.1)
West	403	(35.1)
Residence		
Urban	407	(35.5)
Rural	740	(64.5)
Monthly income per capita in USD (Median [IQR])^b^	190	(46, 243)
Below poverty line (Yes)^c^	223	(19.4)
Insurance		
None	40	(3.5)
Urban employee basic medical insurance	114	(9.9)
Urban residence basic medical insurance	116	(10.1)
New rural cooperative medical scheme	864	(75.3)
Others	13	(1.1)
Direct medical costs	608.7	(286.1, 1301.8)
Costs((Median [IQR])		
Direct non-medical costs	160.5	(74.4, 315.2)
Indirect costs	70.4	(24.6, 296.2)

*SD* Standard deviation, *IQR* Interquartile range, *USD* United States Dollars, *CDC* Centre for disease control and prevention, *HIV* Human immunodeficiency virus

^a^Drug-susceptible pulmonary tuberculosis

^b^A currency exchange rate of CNY 687 to USD 100 (December 2018)

^c^Poverty line in China is Annual per capita household income less than USD 430

### Equity in the distribution of costs

**Fig. 1:**
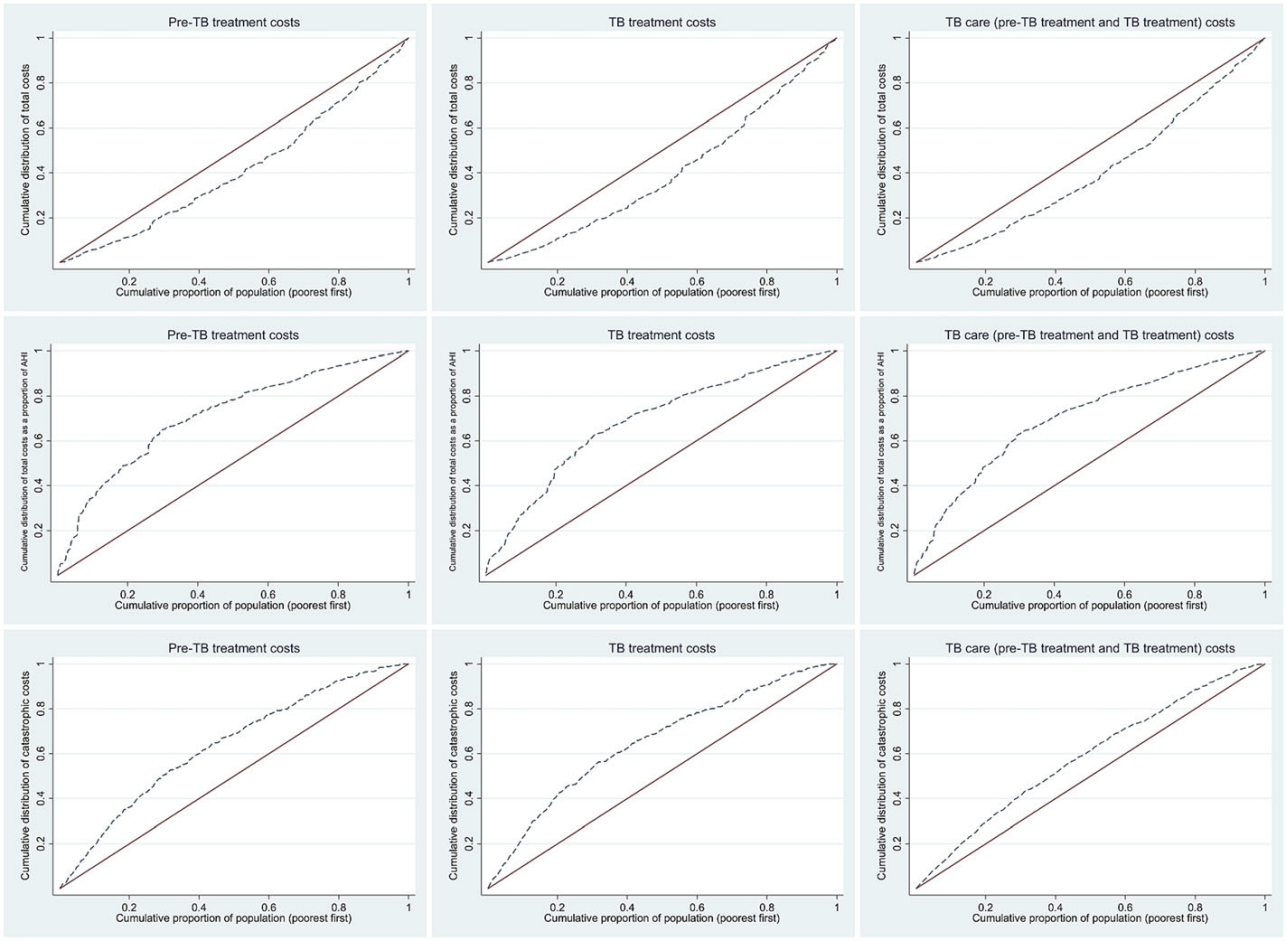
Concentration curves for total costs, total costs as a proportion of pre-TB annual household income and catastrophic costs due to TB care, among TB* affected households in China (2017) (*n =* 1147). TB: Tuberculosis; AHI: Pre-TB annual household income. *drug susceptible TB patients - China’s TB patient cost survey (2017). **all concentration indices were significantly away from the line of equality (*P* <  0.001)

The median (IQR) costs due to pre-treatment, treatment and overall TB care were USD 283.5 (41.8, 945.7), USD 413.1 (231.9, 927.8) and USD 965.5 (461.8, 2059.3), respectively. Total costs due to pretreatment and treatment care were significantly (*P* <  0.001) highest among the richest quintile, while the total costs expressed as a proportion of the AHI and catastrophic costs were significantly (*P* <  0.001) higher among the poorest quintile when compared to the richest quintile (Table 2). This pattern was also reflected in the concentration curves and indices (Fig. 1, Table 3).

**Table 2:** Distribution of total costs and total costs as a proportion of AHI across income quintiles among TB^a^ affected households in China (2017) (*n =* 1147)

Characteristic	Pre-TB treatment		TB treatment		TB care overall	
	Median	(IQR)	Median	(IQR)	Median	(IQR)
Total costs(USD)						
1st MIPC quintile	219.5	(25.2, 579.9)	254.7	(161.3, 490.3)	588.4	(320.6, 1106.7)
2^nd^MIPC quintile	213.8	(9.6, 669.2)	302.7	(193.9, 624.3)	668.5	(331.8, 1590.1)
3^rd^MIPC quintile	220.4	(37.7, 857.3)	425.5	(270.0, 1016.7)	1093.0	(503.3, 1873.0)
4th MIPC quintile	416.4	(103.4, 1376.2)	567.2	(316.3, 1115.8)	1264.4	(661.8, 2781.2)
5th MIPC quintile	438.7	(65.2, 1436.9)	600.5	(367.3, 1378.3)	1638.5	(658.6, 3077.2)
Overall	283.5	(41.8, 945.7)	413.1	(231.9, 927.8)	965.5	(461.8, 2059.3)
*P*-value	< 0.001		< 0.001			< 0.001
Total costs as proportion of AHI						
1st MIPC quintile	30.6	(1.6, 83.5)	33.4	(16.3, 80.1)	72.5	(31.7, 189.8)
2nd MIPC quintile	6.8	(0.0, 28.3)	14.1	(8.9, 32.6)	31.7	(13.7, 68.9)
3rd MIPC quintile	7.7	(1.2, 23.6)	10.5	(5.2, 20.9)	23.5	(11.8, 40.0)
4th MIPC quintile	5.4	(0.9, 15.1)	8.4	(4.6, 17.3)	19.5	(8.5, 37.9)
5th MIPC quintile	2.6	(0.4, 8.6)	5	(2.8, 13.0)	13.5	(5.4, 25.1)
Overall	6.3	(0.6, 25.1)	11.8	(5.2, 27.2)	24.7	(11.3, 60.7)
*P*-value	< 0.001		< 0.001			< 0.001

*TB* Tuberculosis, *AHI* Annual household income, *MIPC* Monthly income per capita, *IQR* Inter quartile range

^a^Drug susceptible TB patients - China’s TB patient cost survey (2017)

**Table 3:** Concentration indices for total costs, total costs as a proportion of pre-TB annual household income and catastrophic costs due to TB care, among TB^a^ affected households in China (2017) (*n =* 1147)

Costs due to TB care	Concentration index (95% *CI*)^*^		
	Total costs	Total costs as proportion of AHI	Catastrophic costs
Pre-TB treatment	0.172 (0.113, 0.231)	−0.429 (− 0.528, − 0.331)	− 0.277 (− 0.327, − 0.227)
TB Treatment	0.199 (0.140, 0.259)	− 0.377 (− 0.449, − 0.305)	− 0.306 (− 0.351, − 0.261)
TB care (pre-TB treatment and treatment)	0.186 (0.145–0.228)	−0.402 (− 0.466, − 0.338)	−0.169 (− 0.197, − 0.141)

*TB* Tuberculosis, *AHI* Pre-TB annual household income

^*^All concentration indices were significantly away from the line of equality (*P* < 0.001)

^a^Drug susceptible TB patients - China’s TB patient cost survey (2017)

### Equity in the distribution of catastrophic costs

**Fig. 2:**
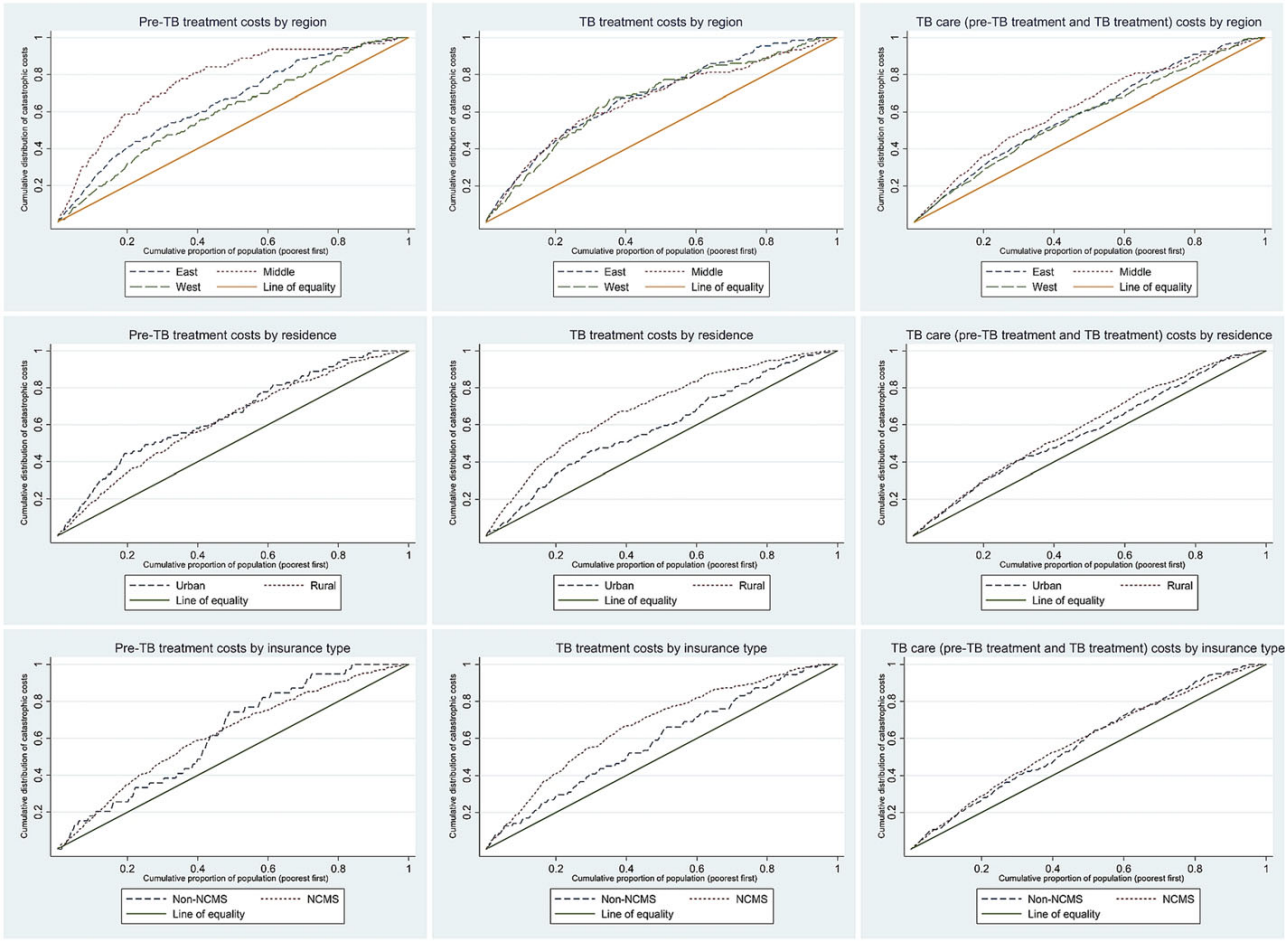
Comparison of concentration curves for catastrophic costs due to TB care among TB* affected households in China (2017): stratified by region, residence and insurance type (*n =* 1147). TB: Tuberculosis; NCMS: new cooperative medical scheme; non-NMCS: include urban employee basic medical insurance, urban residence basic medical insurance, public service medical insurance and other private medical insurance. *drug susceptible TB patients - China’s TB patient cost survey (2017)

All the concentration curves for catastrophic costs stratified by region, area of residence and type of insurance exhibited a pro-poor pattern with statistically significant (*P* <  0.01) concentration indices (Fig. 2, Table 4). The curve of the middle region exhibited statistically significant dominance over the east and west during pre-TB treatment. For catastrophic costs due to treatment, the rural curve dominated over the urban curve while the NCMS dominated the non-NCMS curve in being significantly more pro-poor **(**Table 4**)**.

**Table 4:** Comparison of concentration indices for catastrophic costs due to TB care among TB^a^ affected households in China (2017): stratified by region, residence and insurance type (*n =* 1147)

Variable	Pre- TB treatment		TB Treatment		TB care (pre-TB treatment and treatment)	
	Concentration index (95% *CI*)	Dominance test	Concentration index (95% *CI*)	Dominance test	Concentration index (95% *CI*)	Dominance test
Region						
East	−0.281 (− 0.351, − 0.21)	East versus west – non-dominance	−0.37 (− 0.443, − 0.297)	Non-dominance	−0.182 (− 0.224, − 0.141)	Non-dominance
Middle	− 0.439 (− 0.559, − 0.32)	Middle dominates east as well as west^b^	−0.32 (− 0.393, − 0.247)		−0.228 (− 0.284,-0.172)	
West	−0.197 (− 0.281, − 0.113)		−0.355 (− 0.438, − 0.272)		−0.164 (− 0.212, − 0.114)	
Residence						
Urban	−0.301 (− 0.410, − 0.191)	Non-dominance	−0.174 (− 0.257, − 0.09)	Rural dominates urban^b^	−0.131 (− 0.184, − 0.078)	Non-dominance
Rural	− 0.237 (− 0.292, − 0.181)		−0.383 (− 0.435, − 0.331)		−0.179 (− 0.212, − 0.147)	
Insurance						
NCMS	−0.244 (− 0.297, − 0.192)	Non-dominance	−0.343 (− 0.392, − 0.294)	NCMS dominates non-NCMS^b^	−0.168 (− 0.199, − 0.138)	Non-dominance
Non-NCMS	− 0.25 (− 0.415, − 0.085)		−0.168 (− 0.280, − 0.055)		−0.162 (− 0.233, − 0.091)	

*TB* Tuberculosis, *CI* Confidence interval, *NCMS* New cooperative medical scheme, *Non-NMCS* include urban employee basic medical insurance, urban residence basic medical insurance, public service medical insurance and other private medical insurance

^a^Drug susceptible TB patients - China’s TB patient cost survey (2017)

^b^Statistically significant

## Discussion

Our study revealed that while there is equity in costs due to pre-treatment and treatment care in China, there is inequity in the distribution of catastrophic costs which was also consistently seen across various population sub-groups. Catastrophic costs due to pre-TB treatment were more inequitably shared by the poor in the middle compared to their counterparts in the west and east regions of China. The distribution of the catastrophic costs due to TB treatment was significantly more inequitable among the rural population compared to urban and among those covered under NCMS compared to those covered under non-NCMS insurance schemes.

### Interpretation of key findings

Pro-rich distribution of total costs may be due to the nature of facilities and the type of care availed by the rich; these are different from that sought by the poor. Their capacity to pay is naturally higher than the poorer quintiles, who may not be availing services that are beyond their spending capacity and thus spending lesser than the rich. Another reason could be that the poor are availing schemes by virtue of belonging to poorer socioeconomic status which offer them subsidized or free services. Thereby, the total costs experienced by the poor are lesser than that of the rich.

The poor, however, bore an unfair share of the burden of the total costs expressed as a proportion of AHI and the catastrophic costs. Though they were spending less in absolute quantities, even that took a toll by robbing a significant proportion of the AHI, leading to a financial catastrophe.

The uniform pro-poor distribution of the catastrophic costs due to TB treatment across all population sub-groups studied was significantly more inequitable in the rural areas compared to the urban areas. Rural populations’ access to appropriate, affordable TB services is unsatisfactory compared to that of urban population of China and this difference is exaggerated among the poorer quintiles [13]. Despite the provision of fully subsidized care, patients with TB in China are charged for various reasons like additional investigations and supplements, irrespective of their capacity to pay [14]. Li et al. have reported that a significant proportion of the patients experience catastrophic non-medical expenses [15].

Similarly, the NCMS covered population experienced a more inequitable distribution of catastrophic costs due to treatment compared to those covered by other schemes. This could be a reflection of the rural urban pattern given that the NCMS covers the rural population of China. It has been previously proven that the NCMS did not do much to remove the inequity in the distribution of the TB care costs [9]. Increase in insurance coverage and the reimbursement of expenses has not been translated into reduction in catastrophic costs due to TB care [16]. Various reasons have been attributed to this including that costs incurred as an outpatient are not covered under the NCMS. TB diagnosis and treatment mostly happens in the out-patient settings, thus leaving the costs uncovered. Further, the risk pooling being at the county level and not above doesn’t support high reimbursement rates. Thus, despite over 90% of the rural population being enrolled under NCMS, the benefits drawn by patients with TB are limited.

The middle region showed a significantly more pro-poor distribution of catastrophic costs due to pre-TB treatment care compared to the East and West. This could be due to the differential experience of costs between rich and poor of the respective regions.

### Implications for policy and practice

By 2035, even with aggressive expansion of TB services, catastrophic costs would reduce only by 5–20% when compared to 2015 [17]. Therefore, countries need to move towards attaining universal health coverage and social protection. Universal health coverage will reduce the direct medical costs and social protection will reduce direct non-medical and indirect costs [18–22].

Under universal health coverage, the social insurance schemes in China only marginally reduced catastrophic costs with no effect of inequity [6, 9, 23]. Risk pooling at a level higher than the county, raising the “height” of the NCMS by modifying the benefit package and alternate provider payment mechanisms are recommended [6, 9]. Regulation of unnecessary prescription of additional medications like supplements may also cut costs. The pre-treatment catastrophic costs could be controlled by adhering to standardized diagnosis and treatment algorithms for all forms of TB. This would cut down unnecessary consultations, investigations and associated indirect costs for a patient before she/he is initiated on treatment.

For social protection, TB-specific approach (cash transfers for households with a confirmed case of TB) are expected to be more effective and affordable than a TB-sensitive approach (cash transfers for households with high TB risk to strengthen their economic resilience) [24]. India has started direct benefit transfer of about USD 8 per month up to treatment completion for all patients notified with TB (TB-specific approach) [25, 26].

### Strengths and limitations

To the best of our knowledge, this is the first nationally representative study reporting a detailed analysis of inequity in pre-TB treatment; treatment and overall TB care costs globally. The nationally representative patient level data was collected using the WHO recommended TB patient cost survey guidelines [4]. This equity analysis can be readily adopted to similar nationwide exercises in the world (Viet Nam, Ghana and Indonesia) [27–29].

There were some limitations. Some patients may not have accurately remembered the exact amount they spent for seeking TB care. We attempted to minimize recall limitation by surveying patients still on treatment and imputing costs to the entire episode assuming that all patients complete treatment. However, this might overestimate the costs considering some patients might have failed treatment or been lost to follow up. On the other hand, as we did not include multi-drug resistant TB patients, costs could be an underestimate. Data on service utilization, service quality and outcome were not collected and beyond the scope of this study. The lower costs among poorest quintile may also be due to non-receipt of care.

## Conclusions

We found inequity in distribution of catastrophic costs due to TB care, including pre-treatment and treatment care, in China. This inequity was consistently seen across various population sub-groups. However, inequity was significantly high during treatment phase in rural areas that are covered by NCMS and during the pre-treatment phase in middle region of China. Attainment of universal health coverage and social protection ably complemented by quality TB care is the need of the hour to reduce inequitable distribution of catastrophic costs due to TB care in China.

## Additional files

Additional file 1: Multilingual abstracts in the five official working languages of the United Nations. (PDF 571 kb)

Additional file 2: Annex 1. The parameters used for sample size calculation under in China’s TB* patient cost survey (2017). Annex 2. The per capita gross national product (GNP) of six provinces sampled in China’s TB* patient cost survey (2017). Annex 3. Multi-stage stratified cluster sampling adopted in China’s TB* patient cost survey (2017). Annex 4. The 22 counties sampled in China’s TB* patient cost survey (2017). Annex 5. The questionnaire used in China’s TB* patient cost survey (2017). (DOCX 82 kb)

## Data Availability

The dataset and codebook used in this study are available on request from the corresponding author (zhanghui@chinacdc.cn, huizhang1974@126.com).
