# Supplementary material for: Inequity in catastrophic costs among tuberculosis-affected households in China
Source: Infect Dis Poverty. 2019 Jun 19;8:46. doi: 10.1186/s40249-019-0564-2 (PMC6582572; doi:10.1186/s40249-019-0564-2)
Supplement: Supplementary file 1 — Multilingual abstracts in the five official working languages of the United Nations. (PDF 571 kb) [file 40249_2019_564_MOESM1_ESM.pdf]

Translation of the abstract into the five official working languages of the United Nations

عدم المساواة بين الأسر المصابة بمرض السُلّ في الصين في تكاليف الكوارث المفروضة عليهم

Cai-Hong Xu, Kathiresan Jeyashree,Hemant Deepak Shewade, Yin-Yin Xia, Li-Xia Wang,Yan Liu, Hui Zhang, Li Wang

تمهيد

المعلومات الأساسية: ثمة عدد محدود من الدراسات الوطنية المُقدمة عالميًا خلال فترة القضاء على مرض السُلّ فيما بعد عام 2015، والتي تتعلق بعدم المساواة المرتبط بالثروة المالية في توزيع تكاليف الكوارث الناتجة عن رعاية مرضى السُلّ. بموجب االبرنامج القومي الصيني لمحاربة مرض السُل، تمثل هدفنا في تقييم مدى المساواة في توزيع التكاليف الإجمالية لرعاية مرضى السُل واللتي تتضمن التكاليف الإجمالية وتكاليف العلاج وما قبله، بالإضافة إلى تقييم التكاليف كنسبة لدخل الأسرة السنوي؛ ووصف ومقارنة المساواة في توزيع تكاليف الكارثة التي تشمل التكاليف الإجمالية وتكاليف العلاج وما يسبقه بين الفئات السكانية الفرعية.

المنهجية المُتبعة: دراسة تحليلية متعددة القطاعات يتم استخدام البيانات الموضحة في استقصاء حول التكلفة القومية للقضاء على مرض السُل والذي تم فى عام 2017 في 22 دولة من ست مقاطعات في الصين. وأيضًا، تشمل الدراسة الأشخاص المصابين بالسُل والحساسية من أدوية أمراض الرئة المسجلين بالبرنامج والذين تلقوا علاج مرحلة المرض الحادة لمدة أسبوعين على الأقل. وُصف معدل المساواة باستخدام منحنيات ومؤشرات تركيزية قُورنت باستخدام اختبار سيادة.

النتائج: تمثل متوسط التكلفة الخاصة ب1147 مريض والمتعلقة بمرحلة ما قبل العلاج والعلاج والرعاية الكلية في 283,5 و413,1 و965,5 دولار أمريكي، على التوالي. كما تحملت الشرائح المجتمعية الأغنى تكاليف للعلاج وما قبله أعلى بكثير من الشرائح الأكثر فقرًا؛ حيث أن توزيع تكاليف الكوارث المتناسب مع الدخل السنوي للأسر كان لصالح الشرائح المجتمعية الفقيرة في توزيع التكاليف الإجمالية و تكاليف مرحلتي العلاج وما قبله. كما قُسمت جميع المنحنيات التركيزية الخاصة بتكاليف الكارثة المتضمنة لتكاليف مرحلتي العلاج وما قبله وتكاليف الرعاية الإجمالية تبعًا للمنطقة الشرقية والوسطى والغربية، ومنطقة الإقامة؛ سواء كانت حضرية أو ريفية، بالإضافة على نوع التأمين سواء كان النظام الطبي الحضري التعاوني الحديث أم غيره، وتعرض تلك المنحنيات منوالًا خاصًا لصالح الشرائح الفقيرة ومُحدد إحصائيًا بالمؤشرات التركيز($p < 0.01$). وارتفعت نسبة عدم المساواة في توزيع تكاليف الكارثة الخاصة بعلاج مرض السُل بين مرضى الشرائح الفقيرة الحضرية بشكلٍ كبيرٍ مقارنةً بمرضى الريف، وأيضًا، بين الأشخاص المستفيدين من النظام الطبي الحضري التعاوني الحديث من عدمه.

الاستنتاج: ثمة عدم مساواة في توزيع تكاليف الكارثة الخاصة برعاية مرضى السُل. كما يلزم تحقيق التغطية الصحية الشاملة واستراتيجيات الحماية المجتمعية المُستكملة بواسطة رعاية مضى السُل لتقليل نسبةعدم المساواة في توزيع تكاليف الكارثة الخاصة برعاية مرضى السُل في الصين.

Translated from English version into Arabic by Mona Gamal, proofread by Ahmed Ibrahim, through

TRANSLATORS WITHOUT BORDERS

中国结核病家庭灾难性支出公平性研究

徐彩红, Kathiresan Jeyashree,Hemant Deepak Shewade,夏愔愔,王黎霞,刘艳,张慧,王丽

摘要

引言：在终止结核病时期，全球范围内关于结核病导致的灾难性支出公平性的国家代表性研究非常有限。本研究在中国国家结核病防治规划框架下，评价了结核病花费（包括治疗前花费、治疗期间以及全疗程），结核病花费占家庭年均收入的比例，描述和比较了灾难性支出在不同亚群的公平性。

方法：分析性横断面研究。数据来自 2017 年全国 6 省 22 个县区开展全国结核病经济负担调查。研究对象为国家结核病专报系统登记的接受治疗大于 2 周以上的药物敏感肺结核。采用浓度曲线和浓度指数描述公平性。

结果：纳入调查的 1147 例患者治疗前、治疗期间以及全疗程花费的中位数分别为 283.5 美元、 413.1 美元和 965.5 美元，分布在高收入五分之一的人群治疗前花费和治疗花费显著高于低收入的人群。在治疗前和治疗期间花费占家庭年均收入的比例以及灾难性支出在贫困患者中都偏高。所有灾难性支出（治疗前、治疗期间以及全疗程）按照地区（东、中、西）、居住地（城市、农村）以及医保类型（新农合、非新农合）分层的浓度曲线均显示显著的贫困倾向模式。在治疗期间的灾难性支出贫困倾向模式在农村和城市、新农合和非新农合之间存在显著性差异。

结论：结核病诊疗导致的灾难性支出分布不公平。通过实施高质量结核病关怀的全面健康覆盖和社会保护措施对于降低中国由于结核病导致的灾难性支出分布的不公平至关重要。

Translated from English version into Chinese by Cai-Hong Xu

Distribution inéquitable des coûts catastrophiques parmi les ménages touchés par la tuberculose en Chine

Cai-Hong Xu, Kathiresan Jeyashree, Hemant Deepak Shewade, Yin-Yin Xia, Li-Xia Wang, Yan Liu, Hui Zhang, Li Wang

Résumé

Contexte : Il n'existe, à l'échelle mondiale, qu'un nombre limité d'études représentatives de la situation nationale sur la période faisant suite à la campagne « END TB » de 2015 contre la tuberculose et qui concernent les inégalités liées aux moyens financiers dans la distribution des coûts catastrophiques résultant de la prise en charge de la tuberculose. Dans le contexte du programme national de lutte contre la tuberculose mis en place par la Chine, nous avons voulu évaluer, d'une part, l'équité de distribution du coût total de la prise en charge de la tuberculose (procédures en amont du traitement, traitement et prise en charge globale) et, d'autre part, le pourcentage du revenu annuel des ménages que représente ce coût. Cette évaluation a été complétée par une description et une comparaison de la distribution des coûts catastrophiques (en amont du traitement, traitement et prise en charge globale) entre les sous-groupes de la population.
Méthodes : Étude transversale analytique basée sur les données de l'enquête nationale réalisée en 2017 dans 22 circonscriptions de 6 provinces chinoises sur le coût supporté par les patients atteints de tuberculose. Les cas de tuberculose pulmonaire pharmacosensible recensés dans le cadre du programme et qui avaient bénéficié d'au moins deux semaines de traitement en phase intensive ont été inclus. Des courbes de concentration ont été utilisées pour représenter les inégalités et des tests de dominance pour comparer les indices de concentration.
Résultats : Sur 1147 patients, le coût médian des procédures en amont du traitement était de 283,5 USD, celui du traitement de 413,1 USD et celui de la prise en charge globale de 965,5 USD. Les quintiles les plus riches supportaient des coûts avant et pendant le traitement significativement plus élevés que les quintiles plus pauvres. La distribution des coûts en pourcentage des revenus annuels des ménages et celle des coûts catastrophiques étaient favorables aux plus défavorisés en ce qui concerne la prise en charge globale, ainsi que pour les phases en amont du traitement et pour le traitement. Toutes les courbes de concentration des coûts catastrophiques (en amont du prétraitement, traitement et prise en charge globale) stratifiés par région (est, centre et ouest), habitat (urbain, rural) et type d'assurance (nouveau système médical rural coopératif [NCMS], autres systèmes) montraient également une tendance favorable aux catégories les plus défavorisées, avec des indices de concentration statistiquement significatifs (*P* < 0,01). Cette répartition favorable aux plus défavorisés des coûts catastrophiques du traitement contre la tuberculose faisait apparaître des inégalités significativement plus importantes parmi les patients des zones rurales que dans les zones urbaines, et parmi les bénéficiaires du NCMS par rapport aux autres assurés.
Conclusions : Il existe des inégalités dans la distribution des coûts catastrophiques liés à la prise en charge de la tuberculose. Une couverture médicale universelle et des stratégies de protection sociale, complétées par des soins antituberculeux de qualité, sont essentielles pour réduire ces inégalités en Chine.

Translated from English version into French by Cendrine Strevens, proofread by Suzanne Assenat, through

TRANSLATORS WITHOUT BORDERS

Диспропорции в катастрофических расходах среди семей Китая, затронутых туберкулезом

Цай Хун Сюй, Катиресан Джейяшри, Гемант Дипак Шевад, Инь-Инь Ся, Ли Ся Ван, Янь Лю, Хуэй Чжан, Ли Ван.

Аннотация.
Общие сведения: Имеется лишь ограниченное количество национальных репрезентативных исследований после эпохи end TB 2015 года, касающихся диспропорции в распределении катастрофических расходов, связанных с лечением туберкулеза. В рамках Национальной программы Китая по борьбе с туберкулезом, мы стремились оценить насколько равномерно распределяются все расходы на медицинское обслуживание, связанные с лечением туберкулеза (обследование, лечение и общие затраты), и затраты в соотношении с годовым доходом семьи, а также описать и сравнить справедливость распределения катастрофических расходов (обследование, лечение и общие затраты) среди всех подгрупп населения.
Методы: Аналитическое поперечное исследование, использующее данные национального анализа расходов больных туберкулезом, было проведено в 22 уездах шести провинций Китая. В исследование были включены больные легочным туберкулезом, поддающимся медикаментозному лечению, зарегистрированные в программе, которые прошли как минимум двухнедельную фазу лечения. Суммы были представлены кривыми изменения концентраций, и индексы концентраций сравнивались посредством проверки на доминирующее положение.
Результаты: Из 1147 пациентов, медианы цен на обследование, лечение и общие затраты составляли, соответственно, 283,5, 413,1 и 965,5 доллара США. Пациенты из более богатых квинтилей понесли значительно большие расходы на обследование, лечение и общие затраты, по сравнению с менее богатыми. В целом распределение расходов, в пропорциональном соотношении с годовым доходом семьи и катастрофическими расходами, было значительно смещено в интересах малоимущего населения, как и на стадиях обследования и лечения. Все кривые изменения концентраций, построенные для катастрофических расходов (на обследование, лечение и общий уход), разбитые по регионам (восточный, центральный и западный), местам проживания (городским и сельским районам) и видам страховки (Новая кооперативная система сельского медицинского обслуживания, обеспечивающая сельских жителей (NCMS), страховка вне системы NCMS), так же

продемонстрировали картину ориентации на интересы малоимущих со статистически значимыми индексами концентрации (P < 0,01). Распределение катастрофических расходов, связанных с лечением туберкулеза, в интересах малоимущего населения, было значительно менее равномерным среди сельских пациентов по сравнению с городскими, а также среди клиентов NCMS по сравнению с клиентами, не застрахованными программой NCMS.
Выводы: Наблюдается диспропорция в распределении катастрофических расходов, связанных с лечением туберкулеза. Всеобщий охват услугами здравоохранения и стратегии социальной защиты, подкрепленные качественными услугами по борьбе с туберкулезом, крайне важны для уменьшения диспропорции в распределении катастрофических расходов, связанных с лечением туберкулеза в Китае.

Translated from English version into Russian by Yuliana Boyer, proofread by Michael Orlov, through

La desigualdad en los gastos catastróficos en los hogares afectados por la tuberculosis en China

Cai-Hong Xu, Kathiresan Jeyashree, Hemant Deepak Shewade, Yin-Yin Xia, Li-Xia Wang, Yan Liu, Hui Zhang, Li Wang

Resumen
Antecedentes: en la era posterior a la Estrategia Fin de la Tuberculosis (TB) de 2015, en el mundo hay pocos estudios representativos a nivel nacional sobre la desigualdad relacionada con la riqueza en la distribución de los gastos catastróficos derivados de la atención de la TB. En el marco del programa nacional de lucha contra la tuberculosis en China, tratamos de evaluar el grado de igualdad en la distribución de los gastos totales de la atención de la TB (tratamiento previo, tratamiento y atención general) y los gastos en proporción a la renta anual por hogar, así como de describir y comparar la igualdad en la distribución de los gastos catastróficos (tratamiento previo, tratamiento y atención general) entre los subgrupos de la población.
Métodos: se llevó a cabo un estudio analítico transversal con datos de una encuesta nacional sobre gastos de pacientes con TB, realizada en veintidós municipios de seis provincias de China en 2017. Se incluyó a los pacientes con TB pulmonar resistente a los fármacos registrados en el programa y que habían recibido al menos dos semanas de terapia en fase intensiva. La igualdad se representó utilizando curvas de concentración, y los índices de concentración se compararon aplicando el criterio de posición dominante.
Resultados: de los 1147 pacientes, el gasto promedio del tratamiento previo, del tratamiento y de la atención general fue de 283,5 USD, 413,1 USD y 965, 5 USD, respectivamente. Los quintiles más ricos incurrieron en gastos de tratamiento previo y tratamiento considerablemente más elevados en comparación con los quintiles más pobres. La distribución de los gastos en proporción a la renta anual por hogar y de los gastos catastróficos fue, en general, a favor de los pobres, al igual que durante las fases de tratamiento previo y de tratamiento. Todas las curvas de concentración de los gastos catastróficos (derivados del tratamiento previo, del tratamiento y de la atención general), estratificados por región (oriental, media y occidental), área de residencia (urbana y rural) y tipo de seguro (nuevo sistema médico cooperativo rural [NCMS, por sus siglas en inglés] y no NCMS), también presentaron un patrón a favor de los pobres, con índices de concentración estadísticamente significativos (P < 0,01). La distribución a favor de los pobres de los gastos catastróficos derivados del tratamiento de la TB fue considerablemente más desigual entre los pacientes rurales en comparación con los urbanos, y entre los beneficiarios del NCMS frente a los no NCMS.
Conclusiones: existe una desigualdad en la distribución de los gastos catastróficos derivados de la atención de la TB. La cobertura sanitaria universal y las estrategias de protección social, junto con una atención de la TB de calidad, son fundamentales para reducir la distribución desigual de los gastos catastróficos derivados de la atención de la TB en China.

Translated from English version into Spanish by Mayra León, proofread by Maria Faure, through
