# Supplementary material for: Inequity in catastrophic costs among tuberculosis-affected households in China
Source: Infect Dis Poverty. 2019 Jun 19;8:46. doi: 10.1186/s40249-019-0564-2 (PMC6582572; doi:10.1186/s40249-019-0564-2)
Supplement: Supplementary file 2 — Annex 1. The parameters used for sample size calculation under in China’s TB* patient cost survey (2017). Annex 2. The per capita gross national product (GNP) of six provinces sampled in China’s TB* patient cost survey (2017). Annex 3. Multi-stage stratified cluster sampling adopted in China’s TB* patient cost survey (2017). Annex 4. The 22 counties sampled in China’s TB* patient cost survey (2017). Annex 5. The questionnaire used in China’s TB* patient cost survey (2017). (DOCX 82 kb) [file 40249_2019_564_MOESM2_ESM.docx]

**Additional file 2: Annex 1. The parameters used for sample size calculation under in China’s TB* patient cost survey (2017)**

We calculated sample size using the formula below.


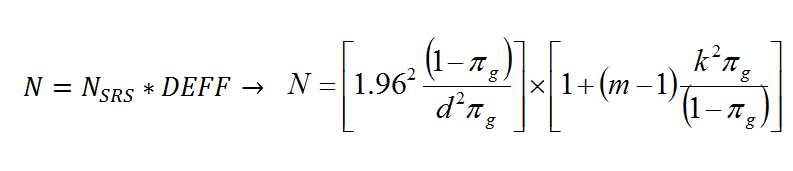


| Parameter | Meaning | Value estimated in this study |
| --- | --- | --- |
| 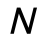 | Number of people included in the patient survey | 977 |
| NSRS | Simple Random Sampling size | 224 |
| DEFF | Design effect | 4.36 |
| 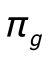 | “Prior guess” of the true proportion of household experiencing catastrophic costs due to TB illness (expressed as a proportion) | 30% |
| 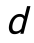 | Relative precision (expressed as a proportion). Recommended 0.20 or 0.25 | 0.2 |
| 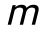 | Cluster size (=number of targeted individuals), assumed to be constant across clusters | 50 |
| 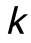 | Coefficient of between-cluster variation. Recommended to assume is in the range 0.4 – 0.6 | 0.4 |

TB – tuberculosis

**Additional file 2: Annex 2. The per capita GNP of six provinces sampled in China’s TB* patient cost survey (2017)**

| Region | Province | GDP per capital in 2016(thousand yuan) | Rank No among 31 province in China |
| --- | --- | --- | --- |
| East | Liaoning | 50 | 14 |
| East | Fujian | 74 | 6 |
| Middle | Henan | 42 | 20 |
| Middle | Hunan | 46 | 16 |
| West | Yun nan | 31 | 30 |
| West | Xinjiang | 41 | 21 |

*GDP- Gross Domestic Product*

**Additional file 2: Annex 3. Multi-stage stratified cluster sampling adopted in China’s TB* patient cost survey (2017)**

| **Step 1** | Two provinces from east/middle/west regions each (six provinces in total) were chosen by simple random sampling. Liaoning and Fujian; Henan and Hunan; and Yunnan and XinJiang respectively. |
| --- | --- |
| **Step 2** | The number of clusters in each stratum was determined, letting them to be proportional to number of TB patients registered in every stratum in the previous year. There were 6 strata in total, which are east urban area, east rural area, middle urban area, middle rural area, west urban area, west rural area. But since the total cluster numbers were limited, probability proportional to size sampling (PPS sampling) was adopted. |
| **Step 3** | A list of NTP’s BMUin the 6 provinces selected in Step 1 was obtained. Each BMU was classified into predominantly rural or urban areas. |
| **Step 4** | According to the lists of BMU created in Step 3 and the number of BMU decided in Step 2, simple random sampling with replacement was done for 6 strata separately |
| **Step 5** | Within sampled BMU facilities, consecutive patients on TB treatment visiting the facility were eligible for inclusion. |

NTP-National tuberculosis programme; BMU- basic management units

**Additional file 2: Annex 4. The 22 counties sampled in China’s TB* patient cost survey (2017)**

| Region | Province | Sites |
| --- | --- | --- |
| East | Liaoning | Da Xin district, Fuxin county, Huanren county, Taian county |
| East | Fujian | Quangang District, Minhou county, ChangLe county, Zhangpu county |
| Middle | Henan | Muye district, Miyang county |
| Middle | Hunan | Lengshuijiang county, Yuetang district, Hongjiang county, Xinning county |
| West | Yun nan | Mile county, Lufeng county, Xiangyun county |
| West | Xinjiang | Yizhou district, A tu shen county, Gaoxin district, Yining District, Zepu county |

**Additional file 2: Annex 5: The questionnaire used in China’s TB* patient cost survey (2017)**

# Baseline survey on household catastrophic health cost due to TB in China in 2017

**Name of Province Name of District**

**Place of interview (facility name)**

**Name of patient; Patient registration number**

**Tel of patient**

**1. Classification of Patient**

**A. Not MDR**

(1)New patient (2) Recurrent patient (3) Return (4)Failed in the Initial Treatment (5) Failure in retreatment

**B. MDR**

(1)New patient (2) Recurrent patient (3) Return (4) Failed in the Initial Treatment (5) Failure in retreatment

**2. Stage of treatment**

(1) Intensive phase (2) Continuation phase (3)Other

**3. Start date of current TB treatment in medical record(year/month/day)**

**4. Start date of treatment in continuation phase if the patient is at treatment stage of continuation phase (year/month/day)**

**4.Date of Interview(year/month/day)**

Introduction to the patient:

My name is (name). The organization I am working for, Chinese Center for Disease Control and Prevention, is interested in the costs that people face when they are treated for TB as well as the costs faced while seeking health care before the diagnosis of TB.

The information that you choose to share will be used for research purposes. It will be shared with other researchers for further analysis and published, but all your personal information will first be deleted in order to ensure full confidentiality.

It is important for you to understand that your participation in this study is completely voluntary. We would be really grateful if you would agree to participate in this study, but do feel free to decline. If you decline, there will be no consequence for you and you will receive all the care and treatment you need at the health facility as usual. If you decline to participate you will not lose any benefit that you are entitled to such as receiving care and support that is provided at the clinic.

If you decide to participate, I would like to stress that you will not receive any reimbursements for the expenses that you report on in this interview.

If you choose to participate in this study, you may still withdraw from the study at any stage without giving any explanation for your withdrawal. Your answers will be kept confidential. At some point I will ask you about your personal income (revenue) and the income of your household. We will NOT provide this information to any tax or welfare authorities, even after the study has been completed.

In charge of this study is the Principal Investigator: (name, address, email). The outcome of this study will be disseminated in an open source journal and you may request a copy from the principal investigator.

This survey will take approximately 20-40 minutes.

**1. Do you want to participate?**

(1)Yes (2) No

**1.1 Because:**

(1) Language not good enough (2) Time constraint (3) Not comfortable (4) Other, specify:

**2.Decision about inclusion or exclusion**

(1) Included (2) Excluded

**3. If excluded, reason for exclusion**

(1) No informed consent (2) Treatment registration group is “other”

**4. Interviewee identity**

(1) Patient (2) Guardian, relationship with patient (3) Other

**Patient signature Date：(y/m/d)**

**Investigator signature Date：(y/m/d)**

# Part I. Patient information of Diagnosis and treatment

**1.Sex: (1)male (2)female**

**2.Birthday: (y/m/d) Age:**

**ID number:**

**3.Annual per capital household income in medical records:**

**4.Date of the first bacteriological TB test**

(1)(y/m/d) (2) Not done or unknown

**5.Bacteriological TB test used and report date:**

(1) Smear microscopy: not done, done-positive, done negative, report date

(2) Culture: not done, done-positive, done negative, report date

(3)Molecular test (such as Xpert MTB/RIF): not done, done-positive, done negative, report date

**6. Date of diagnosis:(y/m/d)**

**7. Diagnosis institution**

(1) General hospitals at county level and above

(2)The Institute of Tuberculosis Prevention and Control at county level and above

(3) CDC at county level and above

(4) Specialized hospital at county level and above

(5) Chinese medicine hospital at county level and above

(6) Township hospital/Community healthcare center

(7)Village clinics/Community Health Station

(8)Private clinics

(9) Other

**8. Date of diagnosis in this facility:(y/m/d)**

**9.Diagnosis institution**

(1) General hospitals at county level and above

(2)The Institute of Tuberculosis Prevention and Control at county level and above

(3) CDC at county level and above

(4) Specialized hospital at county level and above

(5) Chinese medicine hospital at county level and above

(6) Township hospital/Community healthcare center

(7)Village clinics/Community Health Station

(8)Private clinics

(9) Other

**10.Type of TB**

(1) Pulmonary, bacteriologically confirmed

(2) Pulmonary, bacteriologically unconfirmed

**11.Questions about drug susceptibility test**

**11.1 Drug susceptibility test done**

(1) Yes

(2) No/unknown

**11.2 If yes, with what test**

Gene Xpert MTB/Rif (1) yes (2)no

LPA: (1) yes (2)no

Culture with DST: (1) yes (2)no

Other: (1) yes (2)no

**11.3 If yes, drug susceptible results**

(1) Rif-resistant

(2) MDR-TB

(3) Non Rif-resistant/MDR, DR-TB

(4) Non Rif-resistant/MDR, DS-TB

(5) Unknown

**11.4 On MDR-TB treatment**

(1) Yes (2) No

**12. Treatment regimen prescribed**

(1) 2HRZE/4HR

(2) 2HREZS/4HRE

(3) Other first line regimen:___________

(4) Second line standardized regimen: 6 Z Am（Km，Cm）Lfx（Mfx）PAS（Cs，E）Pto /

18 Z Lfx（Mfx）PAS（Cs，E）Pto_

(5) Second line individualized regimen: _____________

Tips：first line anti-TB drugs: INH(H), RFP(R), PZA(Z), EMB(E), SM(S)

Second line anti-TB drugs：kanamycin(K), Amikacin (A), Capreomycin(C), Sodium Aminosalicylate(P), Protionamide(Pto), Cycloserine (Cs), Ofloxacin(Ofx), Levofloxacin(Lfx), Moxifloxacin(Mfx), Clofazimine(Clr), Clofazimine(Cfz), Amoxicillin and Clavulanate(Amx/Clv)

**13. Total duration of planned treatment from start months**

**14. Type of treatment support/supervision; DOT or self-administered treatment?**

(1) DOT

(2)self-administered treatment(skip to question 15)

(3) Not known(skip to question 15)

**14.1 If DOT, who is the current DOT provider/supporter?**

(1) Health facility

(2) Community health worker/volunteer

(3) Workplace

(4) Family member

(5) Other_______________

**15.HIV status**

(1) Positive (2) Negative (3) Not tested (4) Unknown

**16. Results of sputum examination in this institution and report date**

(1) The second sputum: not done, done-positive, done negative, report date

(2) The third sputum: not done, done-positive, done negative, report date

(3) The forth sputum: not done, done-positive, done negative, report date

(4) The fifth sputum: not done, done-positive, done negative, report date

(5) The sixth sputum: not done, done-positive, done negative, report date

(6) The seventh sputum: not done, done-positive, done negative, report date

# Part II. Costs before the current TB treatment (filled for new cases in intensive phase only)

**1. What symptoms did you experience that led you to seek treatment for your current illness/episode:**

(1) [Cough](https://cn.bing.com/dict/clientsearch?mkt=zh-CN&setLang=zh&form=BDVEHC&ClientVer=BDDTV3.5.1.4320&q=%E5%92%B3%E5%97%BD) (2)Expectoration (3)Hemoptysis (4)Asthenia (5)Night sweats

(6)Chest pain (7) Chest tightness (8) Fever (9) Emaciation (10) Other

(11) None(if none, skip to question 3)

**2.The first time you had these above TB symptoms for this TB episode(y/m/d)(skip to question 5)**

**3.If you did not have symptoms, how did you discover may be suffering from tuberculosis?**

(1) Health Examination (2) Active case finding (3) Other

**4. .If you did not have symptoms, when did you find you may suffer from tuberculosis?**

**(y/m/d)**

**5. Time of start treatment in this facility (y/m/d)**

**5.1 Diagnosis institution**

(1) General hospitals at county level and above

(2)The Institute of Tuberculosis Prevention and Control at county level and above

(3) CDC at county level and above

(4) Specialized hospital at county level and above

(5) Chinese medicine hospital at county level and above

(6) Township hospital/Community healthcare center

(7)Village clinics/Community Health Station

(8)Private clinics

(9) Other

**5.2Are you in this agency for the treatment of tuberculosis**

(1) Yes, date of start treatment(y/m/d) and formonths

(2) No.

**6. Before you started your TB treatment at this facility, How many times have you been treated for TBtimes.**

**7.Please describe and fill in the details of the visits before your TB diagnosis**

Table 1 the visits before your TB treatment

| Visit | | | 1^st^ | 2^nd^ | 3^rd^ | 4^th^ | 5^th^ | 6^th^ | 7^th^ | 8^th^ | 9^th^ | 10^th^ |
| --- | --- | --- | --- | --- | --- | --- | --- | --- | --- | --- | --- | --- |
| Type of visit1=outpatient; 2=hospital stay ; 3=emergency | | |  |  |  |  |  |  |  |  |  |  |
| date | | |  |  |  |  |  |  |  |  |  |  |
| Name of facility | | |  |  |  |  |  |  |  |  |  |  |
| Type of provider | | |  |  |  |  |  |  |  |  |  |  |
| Travel time (hour) | | | h | h | h | h | h | h | h | h | h | h |
| Time spent for visit (day, hour) | | | D  H | D  H | D  H | D  H | D  H | D  H | D  H | D  H | D  H | D  H |
| Medical direct payments | Medical payments, total(¥) | |  |  |  |  |  |  |  |  |  |  |
|  | Health insurance reimbursement(¥) | |  |  |  |  |  |  |  |  |  |  |
|  | Out-of-pocket medical payment(¥) | |  |  |  |  |  |  |  |  |  |  |
|  | Registered fee | Total(¥) |  |  |  |  |  |  |  |  |  |  |
|  |  | reimbursement(¥) |  |  |  |  |  |  |  |  |  |  |
|  | Medicine | Total(¥) |  |  |  |  |  |  |  |  |  |  |
|  |  | Reimbursement(¥) |  |  |  |  |  |  |  |  |  |  |
|  | Test | Total(¥) |  |  |  |  |  |  |  |  |  |  |
|  |  | reimbursement(¥) |  |  |  |  |  |  |  |  |  |  |
| Non-medical out-of-pocket payments | Means of transportation | |  |  |  |  |  |  |  |  |  |  |
|  | Travel expenses or round-trip time | | ¥  h | ¥  h | ¥  h | ¥  h | ¥  h | ¥  h | ¥  h | ¥  h | ¥  h | ¥  h |
|  | Food during health care visit or hospital stay(¥) | |  |  |  |  |  |  |  |  |  |  |
|  | Other, including accommodation(¥) | |  |  |  |  |  |  |  |  |  |  |
| overhead expenses | Do you have accompanists, if yes, how many | |  |  |  |  |  |  |  |  |  |  |
|  | Did they lose their working time(1.yes 2.no) | |  |  |  |  |  |  |  |  |  |  |
|  | Income of accompanists(¥) | |  |  |  |  |  |  |  |  |  |  |
| subsidy(¥) | | |  |  |  |  |  |  |  |  |  |  |

Type of provider: (1) General hospitals at county level and above (2) The Institute of Tuberculosis Prevention and Control at county level and above (3) CDC at county level and above (4) Specialized hospital at county level and above (5) Chinese medicine hospital at county level and above (6) Township hospital/ Community healthcare center (7) Village clinics/ Community Health Station (8) Private clinics (9) Other

# Part IV. Cost during current TB/MDR-TB treatment*Unless specified, this section refers to the patient’s current treatment phase only*

***A. Inpatient information***

**1. Are you currently hospitalized?**

(1) Yes (2) No

**2. Have you been previously hospitalized during your current TB treatment phase and because of TB? If yes, how many times?**

(1)Yes.Times (2) No

**3. About how much money and time did you spend for each of these hospitalizations during the current treatment phase?**

Table 2 The money and time spend for each of these hospitalizations

| Hospitalization | | | | | 1^st^ | 2^nd^ | 3^rd^ | 4^th^ | 5^th^ | 6^th^ | 7^th^ | 8^th^ | 9^th^ | 10^th^ |
| --- | --- | --- | --- | --- | --- | --- | --- | --- | --- | --- | --- | --- | --- | --- |
| Date | | | | |  |  |  |  |  |  |  |  |  |  |
| Name of hospital | | | | |  |  |  |  |  |  |  |  |  |  |
| Type of hospital | | | | |  |  |  |  |  |  |  |  |  |  |
| Number of days hospitalized | | | | |  |  |  |  |  |  |  |  |  |  |
| Travel time | | | | |  |  |  |  |  |  |  |  |  |  |
| Medical direct payments | Medical payment(Total) (¥) | | | |  |  |  |  |  |  |  |  |  |  |
|  | Health insurance reimbursement(¥) | | | |  |  |  |  |  |  |  |  |  |  |
|  | Out-of-pocket medical payment(¥) | | | |  |  |  |  |  |  |  |  |  |  |
|  | 1.Bed (¥) | | | |  |  |  |  |  |  |  |  |  |  |
|  | 2.Treatment(¥)(include operation and none-surgical treatment) | | | |  |  |  |  |  |  |  |  |  |  |
|  | 3. Radiography and other imaging | | **Total**(¥) | |  |  |  |  |  |  |  |  |  |  |
|  |  |  | DR(¥) | |  |  |  |  |  |  |  |  |  |  |
|  |  |  | CT(¥) | |  |  |  |  |  |  |  |  |  |  |
|  | 4. Lab tests | | **Total**(¥) | |  |  |  |  |  |  |  |  |  |  |
|  |  |  | Smear(¥) | |  |  |  |  |  |  |  |  |  |  |
|  |  |  | Culture(¥) | |  |  |  |  |  |  |  |  |  |  |
|  |  |  | Molecular test(¥) | |  |  |  |  |  |  |  |  |  |  |
|  | 5. Operation Fee(¥) | | | |  |  |  |  |  |  |  |  |  |  |
|  | 6.Western medicine | First-line anti-TB medicine | | Total(¥) |  |  |  |  |  |  |  |  |  |  |
|  |  |  |  | reimbursement(¥) |  |  |  |  |  |  |  |  |  |  |
|  |  | Second-line anti-TB medicine | | Total(¥) |  |  |  |  |  |  |  |  |  |  |
|  |  |  |  | reimbursement(¥) |  |  |  |  |  |  |  |  |  |  |
|  |  | Other medicine | | Total(¥) |  |  |  |  |  |  |  |  |  |  |
|  |  |  |  | reimbursement(¥) |  |  |  |  |  |  |  |  |  |  |
|  | 7.Chinese medicine (¥) | | | |  |  |  |  |  |  |  |  |  |  |
| Non-medical out-of-pocket payments | Means of transportation | | | |  |  |  |  |  |  |  |  |  |  |
|  | Travel expenses or round-trip time | | | | ¥  h | ¥  h | ¥  h | ¥  h | ¥  h | ¥  h | ¥  h | ¥  h | ¥  h | ¥  h |
|  | Food during health care visit or hospital stay(¥) | | | |  |  |  |  |  |  |  |  |  |  |
|  | Other, including accommodation(¥) | | | |  |  |  |  |  |  |  |  |  |  |
| overhead expenses | Do you have accompanists, if yes, how many | | | |  |  |  |  |  |  |  |  |  |  |
|  | Did they lose their working time(1.yes 2.no) | | | |  |  |  |  |  |  |  |  |  |  |
|  | Income of accompanists(¥) | | | |  |  |  |  |  |  |  |  |  |  |
| subsidy(¥) | | | | |  |  |  |  |  |  |  |  |  |  |

Type of hospital:

(1) General hospitals at county level and above (2) The Institute of Tuberculosis Prevention and Control at county level and above (3) CDC at county level and above (4) Specialized hospital at county level and above (5) Chinese medicine hospital at county level and above (6) Township hospital/ Community healthcare center (7) Village clinics/ Community Health Station (8) Private clinics (9) Other

***B. Outpatient information***

**4.Cost during outpatient visits for medical follow-up (see the doctor or nurse, have tests) and costs of picking up drugs and food costs during ambulatory care in the current treatment phase.**

| Visit | | | 1^st^ | 2^nd^ | 3^rd^ | 4^th^ | 5^th^ | 6^th^ | 7^th^ | 8^th^ | 9^th^ | 10^th^ |
| --- | --- | --- | --- | --- | --- | --- | --- | --- | --- | --- | --- | --- |
| date | | |  |  |  |  |  |  |  |  |  |  |
| Name of facility | | |  |  |  |  |  |  |  |  |  |  |
| Type of provider | | |  |  |  |  |  |  |  |  |  |  |
| Travel time (hour) | | |  |  |  |  |  |  |  |  |  |  |
| Medical direct payments | Medical payments, total(¥) | |  |  |  |  |  |  |  |  |  |  |
|  | Health insurance reimbursement(¥) | |  |  |  |  |  |  |  |  |  |  |
|  | Out-of-pocket medical payment(¥) | |  |  |  |  |  |  |  |  |  |  |
|  | 1.Registered fee(¥) | |  |  |  |  |  |  |  |  |  |  |
|  | 2.Radiography | Total(¥) |  |  |  |  |  |  |  |  |  |  |
|  |  | Radiography(¥) |  |  |  |  |  |  |  |  |  |  |
|  |  | CT(¥) |  |  |  |  |  |  |  |  |  |  |
|  | 3.Lab tests | **Total**(¥) |  |  |  |  |  |  |  |  |  |  |
|  |  | Smear(¥) |  |  |  |  |  |  |  |  |  |  |
|  |  | Culture(¥) |  |  |  |  |  |  |  |  |  |  |
|  |  | Molecular test(¥) |  |  |  |  |  |  |  |  |  |  |
|  | 4.Medicines to treat TB | Total(¥) |  |  |  |  |  |  |  |  |  |  |
|  |  | First-line anti-TB medicine(¥) |  |  |  |  |  |  |  |  |  |  |
|  |  | Second-line anti-TB medicine(¥) |  |  |  |  |  |  |  |  |  |  |
|  | 5.Other medicines(¥) | |  |  |  |  |  |  |  |  |  |  |
|  | 6.Other fee(¥) | |  |  |  |  |  |  |  |  |  |  |
| Non-medical out-of-pocket payments | Means of transportation | |  |  |  |  |  |  |  |  |  |  |
|  | Travel expenses or round-trip time | | ¥  h | ¥  h | ¥  h | ¥  h | ¥  h | ¥  h | ¥  h | ¥  h | ¥  h | ¥  h |
|  | Food during health care visit or hospital stay(¥) | |  |  |  |  |  |  |  |  |  |  |
|  | Other, including accommodation | |  |  |  |  |  |  |  |  |  |  |
| overhead expenses | Do you have accompanists, if yes, how many | |  |  |  |  |  |  |  |  |  |  |
|  | Did they lose their working time(1.yes 2.no) | |  |  |  |  |  |  |  |  |  |  |
|  | Income of accompanists(¥) | |  |  |  |  |  |  |  |  |  |  |
| subsidy(¥) | | |  |  |  |  |  |  |  |  |  |  |

Type of provider:

(1) General hospitals at county level and above (2) The Institute of Tuberculosis Prevention and Control at county level and above (3) CDC at county level and above (4) Specialized hospital at county level and above (5) Chinese medicine hospital at county level and above (6) Township hospital/ Community healthcare center (7) Village clinics/ Community Health Station (8) Private clinics (9) Other

***C. Costs for DOT and food costs during ambulatory care***

**5. On a daily basis, do you currently take your medicines yourself without supervision or support (self-administered) or do you have a treatment supervisor or supporter (DOT)?**

(1) Self-administered(skip to question 15) (2) DOT

**6. If DOT, who is the DOT provider/supporter?**

(1) Health facility (2) Community health worker/volunteer (3) Family member

(4) Other_______________

**7. If DOT,how often?**

(1)once a day (2)once every two days (3)once every three days

(4)never (5)other, specify

**8. If DOT, how long did the last DOT visit take, including travel time and waiting time (total turnaround time)?Minutes.**

**9. What was the cost of transport (return) for the last DOT visit, including parking costs, in total for you and any accompanying household member?**

**10. How much did you spend on food and drinks for the last DOT visit (on the road, while waiting, lunch etc.), in total for you and any accompanying household member?**

**11.Was there a fee paid to the DOT provider?**

(1) Yes. If yes, amount:____________

(2) No

**12.In this treatment phase, do you have accompanists for DOT？How many times?**

(1) Yes, times

(2) No

**13. If yes, did your accompanists lose their working time**

(1) Yes (2) No

**14. If yes, the income of your accompanist¥**

***D. Costs for nutritional/food supplements***

**15. Do you buy any nutritional supplements outside your regular diet because of the TB illness, for example vitamins, meat, energy drinks, or fruits as recommended by health care staff?**

(1) Yes (2) No

**16. If yes, how much did you spend on nutritional supplements in the past week approximately?**

(1)

(2)Not Known(list out the supplements and amount)

# Part V. Influence on patients and their families

***A. Socioeconomic characteristics***

**1. Registered Residence**

(1) Local (2) Other counties in the province (3) Other province

**2. Your local residence time**

(1) Within 3 months (2)3 months to half a year (3)half a year to 1 year (4) more than 1 year

**3.1What is your highest level of education?**

(1)Not attended school

(2) primary school

(3) secondary school

(4) University

(5) graduate school

(6) Other

**3.2what is your marital status**

(1)unmarried (2)married (3)divorce (4)bereft of your spouse

**4. Before TB, are you the highest paid people in your family?**

(1) Yes (2) No

**5. What is the highest level of education the head of the household/primary income earner in the household complete?**

(1) Not attended school

(2) primary school

(3) secondary school

(4) University

(5) graduate school

(6) Other

**6.What is your main occupation?**

(1) School student (2) Teacher (3) Housekeeper or nurse (4) Food and Beverage Industry (5) Commercial service (6) Medical staff (7) Factory worker (8)migrant workers (9)Farmer (10)Herdsman (11)Fisherman (12)Government employee (13)Retiree (14)Homemaker (15)Other (16) Not know

**7. After suffering from tuberculosis, did it bring change to your occupation？**

(1) Yes (2) No(skip to question 9)

**8. If yes, what is your current occupation?**

(1) School student (2) Teacher (3) Housekeeper or nurse (4) Food and Beverage Industry (5) Commercial service (6) Medical staff (7) Factory worker (8)migrant workers (9)Farmer (10)Herdsman (11)Fisherman (12)Government employee (13)Retiree (14)Homemaker (15)Other (16)Not know

**9.Are you suffering from other chronic diseases?(More than one category allowed)**

(1)No

(2) Diabetes

(3) Chronic liver diseases

(4) Chronic Kidney Disease

(5) Anemia

(6) Hypertension

(7) Other

**9.1 Before TB, what is your average monthly medical expense?**

**10. Which kinds of health insurance do you get?**

(1) Urban Employee Basic Medical Insurance

(2) free medical care,

(3) Urban Resident Basic Medical Insurance

(4) New Rural Cooperative Medical Scheme

(5) Commercial health insurance

(6) Other health insurance

(7) None

***B. Income before TB***

- Were you the person who earned the highest income in your household before you contracted TB?

**11. How were you usually paid before you contracted TB?**

(1) Bank transferred salary

(2) Cash

(4) In kind

(4) Cash and in kind

(5) Not paid

**12. How many days a week were you working before you contracted TB?**

**days. And how many hours a day?hours**

**13. How much do you estimate your average net wage or average net revenue from labour related activities (labour income), per month was before you contracted TB?**

**14.How much do you estimate the average revenue from labour(income), after tax, of your household is per month, before you contracted TB?**

***C. Income changes***

**15. How much do you estimate your average net wage or average net revenue from labour related activities (labour income), per month is now?**

**16. How much do you estimate the average revenue from labour(income), after tax, of your household is per month now?**

**17. How many days a week were you working now?days. And how many hours a day?hours**

**18. Approximately how many working days of income have you lost due to your TB illness overall?**

working days before diagnosis of TB (but due to TB disease)

working days after TB diagnosis

**19. Did you or your household receive any social welfare payment after you were diagnosed with TB?**

(1) Yes (2) No(skip to question 21)

**20. If yes, what type and amount (after tax) during the last month?**

Paid sick leave per month, months

Disability grant per month, months

Cash transfer for poor families per month, months

Other cash transfer please specify ,per month, months

**21. Do you currently receive vouchers or goods in kind to cope with TB illness?**

(1) Yes

a. Travel voucher

b. Food support

c. Other, enablers etc.

(2) No(skip to question 23)

**22. From whom do you receive the voucher/ goods.(More than one category allowed)**

(1) Government

(2) NGO

(3) Employer

(4) Private donation

(5) Other, specify

**23. How many adult and children regularly sleep in your house? (including patient, if variable, at time of diagnosis)**

Adult Children

**24. How many rooms are there in the house excluding the bathroom?**

**25.Besides yourself, does anyone else of your household receive treatment for TB? If Yes: How many?**

(1) Yes: person(s) (2) No

**26. Has the TB illness affected your social or private life in any way?**

(1) No

(2) Food insecurity

(3) Divorce or Separated from spouse/partner

(4) Loss of Job

(5) Interrupted schooling

(6) Social exclusion

(7) Other

***D. Coping***

**27.Did you or your household use any savings (cash or bank deposits) to cover costs due to the TB illness?**

(1) Yes (2) No(skip to question 29)

**28. If yes, how much did you use:**

in total

A.before TB treatment started B. Not sure

A.In the intensive phase B. Not sure

A.In the continuation phase B. Not sure

**29. .Did you borrow or receive any money to cover costs due to the TB illness?**

(1) Yes (2) No(skip to question 34)

**30.If yes, how much did you borrow:**

in total

A.before TB treatment started B. Not sure

A.In the intensive phase B. Not sure

A.In the continuation phase B. Not sure

**31. From whom did you borrow? Are you expected to pay the loan(s) back?**

| From whom did you borrow? | Are you expected to pay the loan(s) back? | |
| --- | --- | --- |
| （1）Family | 1）Yes | 2）No |
| （2）Neighbors/friends | 1）Yes | 2）No |
| （3）Private bank | 1）Yes | 2）No |
| （4）Cooperative | 1）Yes | 2）No |
| （5）Employer | 1）Yes | 2）No |
| （6）“Unofficial lender” (Black market) | 1）Yes | 2）No |
| （7）Other, specify | 1）Yes | 2）No |

**32. Have you started paying back the loan? If yes, when did you start?**

(1) Yes, before treatment started

(2) Yes, during the Intensive treatment phase

(3) Yes, during the continuation

(4) No

**33. What is the monthly repayment on the loan, including interest?**

(1) Amountper month, month(s)

(2) I have not started repayment or interest payment

**34. Have you sold any of your property to finance the cost of the TB illness?**

(1) Yes (2) No(skip to question 40)

**35.If yes, what did you sell? (More than one category allowed)**

(1) Land

(2) Livestock

(3) Transport/vehicle

(4) Household item

(5) Farm produce

(6) Gold/jewelry

(7) Other (specify):

**36. If yes, when did you sell property?**

(1) Before treatment started

(2) In the Intensive treatment phase

(3) In the continuation phase

**37. How much money did you receive from the sale of all items of your property?**

in total

A.before TB treatment started B. Not sure

A.In the intensive phase B. Not sure

A.In the continuation phase B. Not sure

**38.The assets that you sold, were they previously supporting the family income (or expenditure)? If yes indicate monthly income previously generated by the assets.**

(1) Yes (amount):__________

(2) No

**39. What is the estimated market value of all the property you sold?**

Value:__________________

**40. Did anyone in your household drop out of school or interrupt schooling to assist the household as a consequence of your TB illness?**

(1) Yes, __________persons

(2) No(skip to question 42)

**41.What were their age and sex and for how long did they drop out?**

(1) Age: Sex: Duration: months

(2) Age: Sex: Duration: months

(3) Age: Sex: Duration: months

**42. On a scale of 1 to 5, in which 1 is no impact and 5 is very serious impact, to what extent has the TB illness affected the household financially?**

(1) 1 = No impact

(2) 2 = Little impact

(3) 3 = Moderate impact

(4) 4 = Serious impact

(5) 5 = Very serious impact

**43. Indicator of family economic and assets**

| Do you have your own house? | (1) Yes (2) No |
| --- | --- |
| If yes, how many houses do you have? | ________ |
| The total area of your house | ________m^2^ |
| Building material of your house | (1)brick-wood (2) brick-masonry (3)civil (4)stone (5)Other________ (6)Not know |
| Building age of the house | (1) (2)Not know |
| Do you have a car? | (1) Yes (2) No |
| Do you have piano or other instrument | (1) Yes (2) No |
| Do you have fitness equipment, like treadmill? | (1) Yes (2) No |
| Do you have a video camera? | (1) Yes (2) No |
| Do you have a computer? | (1) Yes (2) No |
| Do you have a music center? | (1) Yes (2) No |
| Do you have a electric bicycle? | (1) Yes (2) No |
| Do you have a motorcycle? | (1) Yes (2) No |
| Do you have a television? | (1) Yes (2) No |
| Do you have a refrigerator? | (1) Yes (2) No |
| Do you have an air-condition? | (1) Yes (2) No |
| Do you have a washing machine? | (1) Yes (2) No |
| Do you have a water heater? | (1) Yes (2) No |
| Do you have a water fountain? | (1) Yes (2) No |
| Do you have a gas range? | (1) Yes (2) No |
| Do you have a microwave oven? | (1) Yes (2) No |
| Do you have a telephone or mobile phone | (1) Yes (2) No |
| Do you have an agricultural vehicle | (1) Yes (2) No |
| Do you have a large agricultural machinery | (1) Yes (2) No |
| Do you have large livestock | (1) Yes (2) No |
